# The incidence and nature of drug-related hospital admission: A 6-month observational study in a tertiary health care hospital

**DOI:** 10.4103/0976-500X.77095

**Published:** 2011

**Authors:** Harminder Singh, Bithika Nel Kumar, Tiku Sinha, Navin Dulhani

**Affiliations:** *Department of Pharmacology, Govt. Medical College, Jagdalpur, Chhattisgarh, India*; 1 *Department of PSM, Govt. Medical College, Jagdalpur, Chhattisgarh, India*; 2 *Department of Medicine, Govt. Medical College, Jagdalpur, Chhattisgarh, India*

**Keywords:** Adverse drug reactions, drug related problems, over the counter drug

## Abstract

**Objective:**

To assess and evaluate the frequency, severity and classification of drug-related problems (DRP) resulting in hospitalization in an internal medicine department of a large tertiary care hospital and to identify any patient, prescriber, drug, and system factors associated with these events.

**Materials and Methods:**

A prospective and descriptive study carried out in Department of Medicine, Government Medical College, Jagdalpur. The DRP and relevant data were recorded on the personal record of every individual patient, filled during the course of treatment.

**Result:**

A total of 3560 patient’s records were analyzed. Among them118 admissions were due to DRP. The most common DRP noted was noncompliance in part of patient’s i.e 55 (46.6%). Statistically significant correlations were found in the number of prescribed drugs and over the counter drugs (OTC) used by patients.

**Conclusion:**

The DRP that attributed to hospital admission are mostly avoidable through proper patient education and strengthening the need of pharmacovigilance with little more vigilance in patient care.

## INTRODUCTION

The uses of medications are associated with problems that involve a broad set of clinical situations and can result in significant drug-related morbidity and mortality. Over the last 40 years, advances in drug therapy has enhanced patient care but also led to a noticeable increase in the incidence of drug related problems (DRP).[1] Drugs are usually prescribed with the objective of achieving an optimal therapeutic outcome. When the outcome is not optimal, a DRP has occurred.[2,3] Studies in developed countries showed that approximately 5% of all hospital admissions were drug related and 50% of those were avoidable.[4]

There are several predisposing factors for adverse drug reactions (ADR). These include multiple drug therapy, age, sex and polypharmacy, inter current diseases, race and genetic polymorphism.[5] Numerous studies have investigated the problem of drug-related morbidity in ambulatory care, emergency department, and hospitalized patients.[6,7]

The World Health Organization (WHO) defines adverse drug reaction as any noxious, unintended, or undesired effect of a drug occurring at dosages administered in humans for prophylaxis, diagnosis or treatment.[8] This definition, excludes many other classifications of adverse drug events that may result in hospitalization, such as untreated indication, improper drug selection, sub therapeutic or supratherapeutic dosage, noncompliance, drug interaction, and drug use without an indication.[9] We need a more comprehensive, clinically important, and reproducible definition of adverse drug event that would result in a more accurate and meaningful characterization of drug-related hospitalization.

Most of the studies on DRP have been done internationally, so in India very limited research has been conducted to exemplify the impact of DRP that result in hospital admission although the fact is that most of these DRP are avoidable with little vigilant effort.

In this era of increased awareness to improved patient safety coupled with continuing budget restraints, accurate characterization of drug- related hospitalization is an important step towards reducing the potentially significant load that these problems place on our health care system.[10]

The purpose of this study was to prospectively evaluate the frequency, severity, and classification of adverse drug events resulting in hospitalization in an internal medicine department of a large tertiary care hospital, and to identify any patient, prescriber, drug, and system factors associated with these events.

## MATERIALS AND METHODS

This prospective observational study was conducted at department of internal medicine, Government Medical College, Jagdalpur (Chhattisgarh) with the help of department of Pharmacology. Ethics approval was obtained from the Clinical Research Ethics Board and written consent of patients were taken as per ethical guidelines.

All patients who presented to the medicine department during a six months period from January to June 2010 were eligible for enrolment. Inclusion and exclusion criteria: All those cases requiring hospital admission to medicine department due to health problem during a six months period from January to June 2010 were included for the study. Cases which were treated from the Out Patient Departments (OPDs), and which do not require hospital stay, were excluded from the study.

Each patient was interviewed to determine the chief complaint, history of the present illness, past medical history, medication history and allergy status. Information from the physical examination conducted by the treating physician or resident, laboratory results and results of diagnostic tests were used as necessary. Patients were followed up until hospital discharge. Drug related problems were noted according to Hepler and Strand classification of drug-related problems.[11] A drug related hospitalization was defined as drug related if it was directly related to one of eight predefined classifications: adverse drug reaction, drug interaction, improper drug selection, untreated indication, subtherapeutic dosage, supratherapeutic dosage, noncompliance, and drug use without indication.[3] We used the WHO’s definition of adverse drug reaction and included all reactions to drugs administered at appropriate dosages, as well as those associated with abnormal drug concentrations or laboratory values.[3]

Severity was classified as mild (laboratory abnormality or symptom not requiring treatment), moderate (laboratory abnormality or symptom requiring treatment or admission to hospital or resulting in nonpermanent disability), severe (abnormality or symptom that was life-threatening or resulted in permanent disability) or fatal.[12] Drug-related visits were defined as preventable if drug treatment or lack thereof, was inconsistent with current best practice. Such inconsistencies included inappropriate drug, dosage, route or frequency for the patients clinical condition, age, weight or renal function, known drug allergy or previous reaction to drug; known drug interaction, non-adherence, lack of laboratory monitoring and prescribing, dispensing or administration errors.[7,13]

### Data collection

Information related to the patients was collected from the patient files by using a structured questionnaire. Face-to-face enquiries were done with patients along with their attendants and also attending doctors, if required. Proper details and information regarding the matter was discussed with the concerned doctors before entrusting the responsibility. All the available medical records of the patient were studied from the patient’s history. The data were analyzed by using Microsoft Excel in a computer. Data analysis has been presented in tabulated form.

### Statistical analysis

We generated descriptive statistics, specifically means and standard deviations. The primary outcomes of DRP were reported as a percentage with 95% confidence interval (CI). The secondary outcome comparison was carried out using logistic regression method and chi square test to evaluate associations between drug-related visits. P< 0.05 was considered significant.

## RESULTS

During the study period, 3594 patients presented to the medicine department 34 (0.9%) left the medicine department without being seen by physician or resident. Thus, we included 3560 patients (whose demographic characteristics are presented in Table 1) in the final analysis. The mean age of these patients was 49.8 (±18.23) years; 40% were female. Among 3560 admissions 118 were due to drug related problems. The classification of drug related admissions are given in Table 2. The most common drug related problem noted was noncompliance.

**Table 1:** Baseline data of the 3560 hospitalized patients

Variable	Value
Age (Mean ± SD)	49.8 ± 18.23
Number of drug/prescription (Mean ± SD)	4.09 ± 3.3
Number of OTC drugs (Mean ± SD)	2 ± 1.85
Female sex	1424 (40%)
Education below high school	1602 (45%)
Number of patients with low socioeconomic status	1747 (49.07%)

**Table 2:** Classification of the 118 drug related hospital admissions

Drug related classification	Number of hospitalization (%)	95% CI
Noncompliance	55 (46.6)	(37.61 – 55.61)
Adverse drug reactions	26 (22.03)	(14.55 – 29.51)
Supratherapeutic dosage	13 (11.02)	(5.37 – 16.67)
Subtherapeutic dosage	9 (7.63)	(2.84 – 12.42)
Untreated indications	5 (4.24)	(0.6 – 7.88)
Drug use without indication	5 (4.24)	(0.6 – 7.88)
Drug interaction	4 (3.39)	(0.12 – 6.66)
Improper drug selection	1 (0.85)	(-0.81 – 2.51)

Factors associated with drug-related hospital admission (in medicine department) and the comparisons between two populations are shown in Table 3. Severity was classified as mild in 19 cases (16.1%, 95% CI 9.47% to 22.7%), moderate in 91 cases (77.12%, 95% CI 69.54% to 84.70%) and severe in 08 cases (6.78%, 95% CI 2.24% to 11.32%). The few of most common drugs associated with definite DRP were oral hypoglycemic, antihypertensive, chemotherapy (especially anti retroviral agents) and insulin. Most of the definite and possible DRP were definitely preventable. Statistically significant correlations were found in number of prescribed drug and over the counter drug (OTC) used by patients. Interestingly the education status of two populations that is patients with drug related hospitalizations (n=118) and patients without drug related hospitalizations (n=3442) were found statistically significant.

**Table 3:** Factors associated with drug-related hospital admission in medicine department

Variable	Patients with drug related hospitalizations N=118	Patients without drug related hospitalizations N=3442	*P* value
Age (Mean ± SD)	50.5 (17.8)	49.1 (18.6)	>0.05
Number of drug/ prescription (Mean ± SD)	4.9 (3.5)	3.29 (3.1)	<0.001
Number of OTC drugs (Mean ± SD)	2.5 (1.9)	1.5 (1.8)	<0.001
Female	51 (43.2%)	1373 (39.8%)	>0.05
Education below high school	65 (55%)	1537 (44.65%)	<0.05
Patients with low socioeconomic status	61 (51.7%)	1686 (48.9%)	>0.05

## DISCUSSION

In this study, we found that drug related hospital admission accounted for 3.31% medicine department admission among them 78% was considered preventable. Although 77.12% patient outcomes associated with drug-related hospitalization were moderate, 6.78% were considered severe, and 0.7% under sever category resulted in fatal outcome. Number of prescribed drug and use of OTC drugs appeared to be higher among patients whose visits were drug-related than among patients who presented for other reasons. The prospective design of this study and its large sample size increases the likelihood that our estimates of drug-related visits and related factors are accurate. Over the past few years, several published studies have addressed the problem of drug related morbidity in various practice settings.[2,6] Studies on drug-related hospitalization have estimated that approximately 5% to 10% of all hospital admissions are drug related but in our study the percentage is quite low compared to the previous studies, which may be due to the high level of illiteracy, poverty and tribal populated area. So all the related problems have not come to the health care facilities or they may have misconception that these problems are natural course of the diseases.[7,14–16]

Major differences concern our prospective design, in this study; patients were prospectively included on admission and were followed up until discharge. Other prospective studies identified frequencies of fatal medication related hospitalizations of 0.15% to 9%, which are comparable to present study.[12,17] The classification and specific drug therapy associated with hospitalizations in our study are consistent with those in previous reports. Adverse drug reaction, improper drug selection, and noncompliance were the most common reasons of drug-related hospitalization in our study population. Adverse drug reaction and noncompliance have been consistently cited as the primary reasons for drug-related morbidity, regardless of study setting.

The need for direct patient contact by pharmacists and other health care team members is obvious and a collaborative and interdisciplinary patient care model is most beneficial in providing safe and effective therapy.[18] Among all DRP related admissions, non-compliance was the foremost and account for 46.6% of total admission which may have strong correlation with the illiteracy, poverty and misconceptions. Being a tribal populated area the patients started visiting providers of non-conventional therapies and may even stop conventional medications. This was an important reason for noncompliance and had not been reported in previous studies. Complicated medication regimens and inability to recall the regimen and the greater number of preparations used were other important reasons associated with increased risk of non-compliance and increased risk of a hospitalization related to non-compliance.

A factor limiting the scope of this study was the fact that our study involved patients admitted to an internal medicine unit, so a significant number of patients with minor DRP who were not admitted may have been missed from OPD, emergency and other major departments. Future research should focus on interventions to explore the way by which one can ensure higher admission rate among patients with drug-related visits and optimal strategy that may involve interventions outside the hospital to improve prescribing practices and monitoring, particularly among high-risk patients or patients taking high-risk medications or patient on polypharmacy.[19] Our results once again highlight the need of well known principle of pharmacovigilance with simpler regimens with fewer pills to be taken each day as well a well established pharmacy to link between medical practitioner and the patients to make them more vigilant towards the drug they consume and reporting the adverse effects immediately to the concern authority.
